# Effect of Peony-Glycyrrhiza Decoction on Amisulpride-Induced Hyperprolactinemia in Women with Schizophrenia: A Preliminary Study

**DOI:** 10.1155/2017/7901670

**Published:** 2017-11-26

**Authors:** Ping Yang, Liang Li, Dong Yang, Chaoying Wang, Hongli Peng, Huiyong Huang, Xuejun Liu

**Affiliations:** ^1^Department of Psychiatry, Brains Hospital of Hunan Province, Changsha, China; ^2^Provincial Key Laboratory of TCM Diagnostics, Hunan University of Chinese Medicine, Changsha, China

## Abstract

**Objective:**

The aim of this study is to observe the effect of Peony-Glycyrrhiza Decoction (PGD) on hyperprolactinemia in women with schizophrenia induced by Amisulpride.

**Material and Methods:**

A total of 41 female schizophrenia patients receiving Amisulpride were randomly divided into placebo (*n* = 20) and PGD groups (*n* = 21). Maintaining the original Amisulpride dose, the two groups were given placebo and PGD, respectively. The levels of Prolactin (PRL) and other hormones were measured on the initial day and at weeks 4 and 8 after treatment. Changes of clinical symptoms in patients with hyperprolactinemia were observed. The PANSS scores were recorded to assess the psychotic symptoms.

**Results:**

Compared with placebo group, level of PRL decreased while Progesterone increased remarkably in the PGD group at weeks 4 and 8 (*p* < 0.01), and level of Estradiol in the PGD group increased significantly at week 8 (*p* < 0.05). There were no differences in PANSS scores and biochemical indexes between two groups at weeks 4 and 8.

**Conclusion:**

PGD can improve symptoms of hyperprolactinemia and hormone levels in women with schizophrenia caused by Amisulpride, without affecting their mental symptoms and biochemical indexes.

## 1. Introduction

Schizophrenia, a severe mental illness, with high prevalence rate and disability rate, has been characterized by hallucinations, delusional perception, delusions of control, thought disorders, and social function withdrawal [1]. The use of antipsychotic medications is still the main therapeutic method, which can stabilize the patient's mental symptoms, even help them recover social function and return to society [2]. However, the long-term use of antipsychotic medications may lead to a large quantity of adverse reactions, such as the extrapyramidal reaction in the nervous system, leucopenia in the blood system, and Prolactin (PRL) and metabolic disorders in the endocrine system. It will affect the patient's physical health, treatment compliance, and therapeutic effect greatly [3]. If the adverse reactions could not be controlled in time, the patient may have to face the dilemma of drug withdrawal, which subsequently results in disease recurrence [4].

The majority of antipsychotic medications in clinic, such as Haloperidol, Chlorpromazine, Risperidone, Amisulpride, Sulpiride, and Olanzapine, will bring hyperprolactinemia [5]. Some study showed that the prevalence of hyperprolactinemia induced by antipsychotic medications was 42%–89% in schizophrenia patients [6]. In recent years, with the use of Risperidone and Amisulpride, two atypical antipsychotics, the prevalence was as high as 70%–100% [7], and it was even higher in young patients, especially young women [8]. The hyperprolactinemia has been regarded as the most common adverse reaction.

PRL is a polypeptide hormone, synthesized and secreted by luteotroph in the anterior pituitary, and its main function is to regulate reproduction, lactation, and puerpera behavior [9]. Hyperprolactinemia refers to a kind of pathologic status, in which the level of peripheral serum PRL increases continuously [10]. Antipsychotic medications may block the anterior pituitary D2 receptor directly, subsequently weaken the inhibition effect of the dopamine on the secretion of luteotroph, and then cause the ascending of PRL [11]. Nearly all the typical antipsychotics had a strong affinity for D2 receptors. Once they are combined with D2 receptor, the dissociation rate is much too slow, which lead to persistent ascending of PRL. However, atypical antipsychotics have different dissociation rates with D2 receptors, so they differed on the effect of the PRL secretion [12]. Both the parent drug and active metabolite of Risperidone or Amisulpride have a strong effect on peripheral and central dopamine receptors, resulting in hyperprolactinemia [13].

Hyperprolactinemia may cause short-term menstrual disorders, distending pain of the breast, lactation, and sexual dysfunction. If it lasts for a long time, the effect to reproductive development, bone density, and cardiovascular system will come into being [14]. Therefore, the treatment to hyperprolactinemia is of great importance. At present, the first-use drug is Bromocriptine [15], but it will bring depravation of psychiatric symptoms, tremor, and other side effects easily in women with schizophrenia. Risks are more higher than benefits [16]. Studies have also shown that Aripiprazole can significantly reduce the level of PRL induced by Risperidone [17]. However, the combined use of two or more antipsychotic drugs will lead to higher incidence of adverse reaction.

In recent years, the intervention of Chinese herbs in hyperprolactinemia caused by antipsychotic drugs has been studied. The results showed that Peony-Glycyrrhiza Decoction (PGD) could increase the binding capacity of dopamine receptor, inhibit the secretion of PRL [18], and significantly reduce the level of Risperidone-induced PRL [19]. Therefore, in this study, we aimed to observe the clinical effect of PGD on Amisulpride-induced hyperprolactinemia in female schizophrenia patient, then detect the changes of levels of PRL and other related hormones, and assess whether it is safe and reliable or not. The results will not only provide the clinical basis for further study on the mechanism of PGD treating Amisulpride-induced hyperprolactinemia, but also widen the eyesight for the prevention and treatment research of Chinese herbs to the antipsychotics-induced adverse reactions.

## 2. Materials and Methods

### 2.1. Participants and Study Design

In this study, female participants aged 18–40 years with diagnosis of paranoid schizophrenia (F20.0 according to ICD-10 (10th revision of the International Statistical Classification of Diseases and Related Health Problems)) were screened. Majority of return visits were conducted in outpatient clinics; only few were finished in inpatient department. Patients in stable physical, neurological, and endocrinological condition with normal laboratory values (blood tests and biochemical tests, including liver, kidney, and thyroid parameters) were enrolled to the study. Clinical inclusion criteria included (1) taking Amisulpride monotherapy for at least 3 months; (2) stable mental state, stable treatment for at least 3 months, and PANSS scores less than 60 [20]; (3) level of serum PRL higher than 24 ng/mL [21]; (4) at least one overt hyperprolactinemia-related symptom, including amenorrhea, abnormal menstruation, galactorrhea, reduced libido, or sexlessness; (5) screening negative for pregnancy in women and agreeing to use effective contraception methods during the study. The protocol of this study was approved by the medical ethical committee of Brains Hospital of Hunan Province. All participants and their guardians were given the voluntary, written, and informed consent before entering the trial. Patients who had any of the following conditions were excluded from the study: (1) PRL pituitary adenoma, polycystic ovary syndrome, and other serious somatic diseases; (2) a history of neuroleptic malignant syndrome or tardive dyskinesia; (3) suicide ideas or attempts or aggressive behavior; (4) a history of alcoholism and drug abuse in the past 1 year; (5) currently treated with other endocrine drugs, Chinese medicine, and other natural products; (6) a history of allergy to herbal medicine; and (7) lactating.

This is a preliminary study. The double-blind, randomized, placebo-controlled trial, lasting for 2 months, was conducted between January 2014 and February 2017 in the Department of Psychiatry of Brains Hospital of Hunan Province. A total of 41 patients were included in this study. Patients were randomly divided into placebo (*n* = 20) and PGD groups (*n* = 21). The baseline characteristics of patients were tested before this study.

### 2.2. Products

Placebo was provided by Jiuzhitang Co., Ltd., which was made of auxiliary materials, containing no medicine. The appearance and dosage form had no difference from PGD.

**Figure 1:**
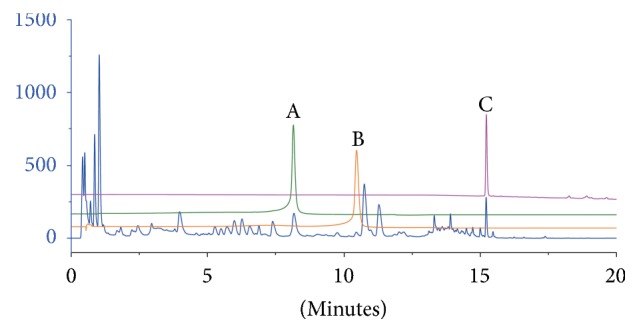
The contents of three major constituents contained in PGD preparation used in this study. A, paeoniflorin; B, liquiritin; C, glycyrrhetinic acid.

PGD was also entrusted to Jiuzhitang Co., Ltd. The standardized preparation of PGD was established in Jiuzhitang Co., Ltd., in compliance with Pharmacopoeia of China [22] and Good Manufacture Practice of Medical Products. Briefly, sliced and broiled Paeoniae Radix Alba (60 mg) and Glycyrrhiza Radix Et Rhizoma (30 mg) in a ratio of 2 : 1 in weight were immersed and boiled in an 8-fold volume of distilled water for 2.5 hours. The placebo and intervention were identical in appearance, taste, and smell. The contents of three major known pharmacologically active constituents of PGD (paeoniflorin, liquiritin, and glycyrrhizic acid) were measured using Ultra Performance Liquid Chromatography (UPLC) and the result was shown in Figure 1. The UPLC profile of the PGD preparation used in this study was highly comparable with those used in previous studies.

### 2.3. Treatments

Participants were randomly allocated to receive either PGD or a placebo twice a day for 8 weeks. They were asked not to alter their antipsychotic medication during the intervention period. This was monitored at monthly visits. A double-blind follow-up visit was conducted 8 weeks after the completion of the treatment. Participants were required to attend biweekly or monthly visits during the study.

During the course of the study, patients were allowed to have concomitant treatment with other psychotropic drugs. These mainly included agents for the treatment of extrapyramidal symptoms and insomnia, for example, Benzhexol Hydrochloride and Benzodiazepines. Adjustment of dosage and addition and termination of psychotropic medication were conducted at the discretion of the participant's psychiatrist, on the basis of the individual. Concomitant use of other herbal and natural products was not allowed during the study period.

### 2.4. Measurements

All measurements were taken three times at the beginning of this study, weeks 4 and 8 after treatment. Three 10 mL blood samples for hormones assay were obtained. Each sample was collected from the cubital fossa veins between 8:00 and 9:00 a.m. before meal and centrifuged at 1000 G for 10 minutes at 4°C. Sera were then separated and stored at −20°C for assay. Serum concentrations of PRL, Estradiol, Progesterone, FSH, and LH were measured using chemiluminescent immunoassay.

In order to ensure the safety of medication, all patients liver and kidney function, blood glucose, blood lipid, and TSH in the initial day and at week 8 were detected. The TESS was evaluated independently by 2 elder attending physicians, and the concordance correlation coefficient > 0.88.

### 2.5. Clinical Assessments

Clinical symptoms of schizophrenia were assessed using PANSS scores and its subscores (positive, negative, and general symptoms) [20] in the initial day and at weeks 4 and 8 after treatment. Record the changes in symptoms, such as amenorrhea, abnormal menstruation, galactorrhea, reduced libido, and sexlessness.

### 2.6. Statistical Analysis

The randomization code was not broken until all data were collected. Statistical analysis was performed using SPSS 24.0. For continuous variables, descriptive statistics (means, standard deviations, and 95% confidence interval) were calculated, while for discrete variables number of patients and percentages are given. Shapiro-Wilk test was used to test normality of distribution, and variables that did not follow normal distribution were transformed for best normality. Means, standard deviations, and confidence intervals are reported for nontransformed variables, and results of tests are reported for transformed or nontransformed variables. The difference between proportions was analyzed by Fisher's exact test, while intergroup differences were assessed by *t*-test. *p* values less than 0.05 were considered as significant and those less than 0.01 were significant remarkably.

## 3. Results

### 3.1. Baseline Characteristics of Patients

The two groups of patients were given placebo and PGD, respectively, lasting for 8 weeks. In the end, 34 of them have completed this study. In the placebo group, 5 patients were lost (25%), among them 2 patients had to quit because of severe akathisia and 3 patients due to amenorrhea after 4 weeks, while in the PGD group only 2 patients were lost (9.52%) for extrapyramidal side effect after 4 weeks. It was a double-blind study. The baseline characteristics of patients are shown in Table 1. No significant differences on baseline variables were observed between these 2 groups.

### 3.2. Levels of PRL and Other Hormones

**Figure 2:**
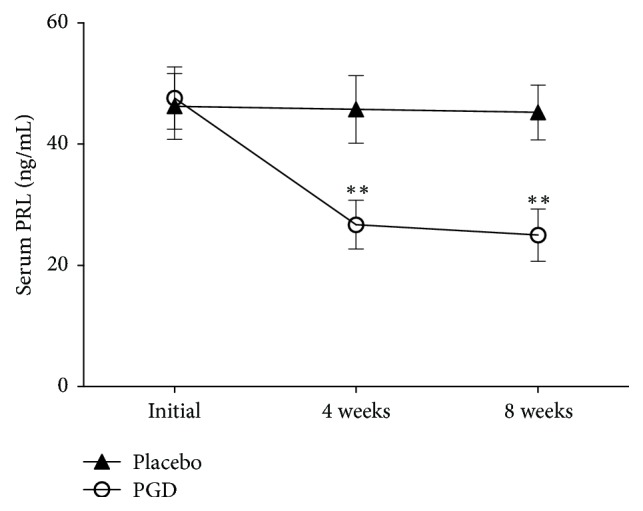
Mean levels of PRL in the study groups. Vertical bars represent standard deviations. Compared with placebo group, ^*∗∗*^*p* < 0.01.

**Figure 3:**
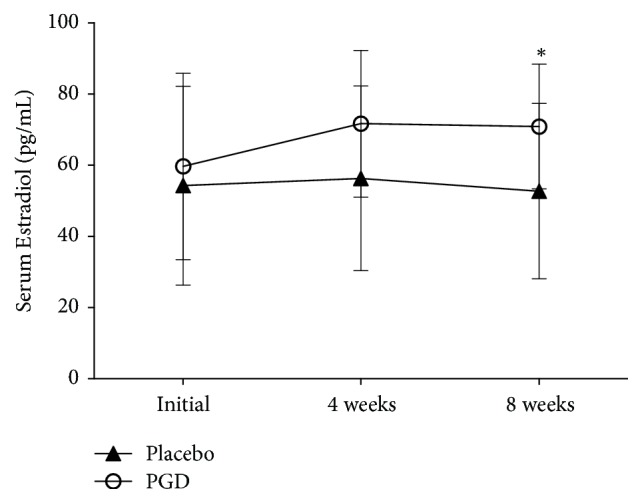
Mean levels of Estradiol in the study groups. Vertical bars represent standard deviations. Compared with placebo group, ^*∗*^*p* < 0.05.

**Figure 4:**
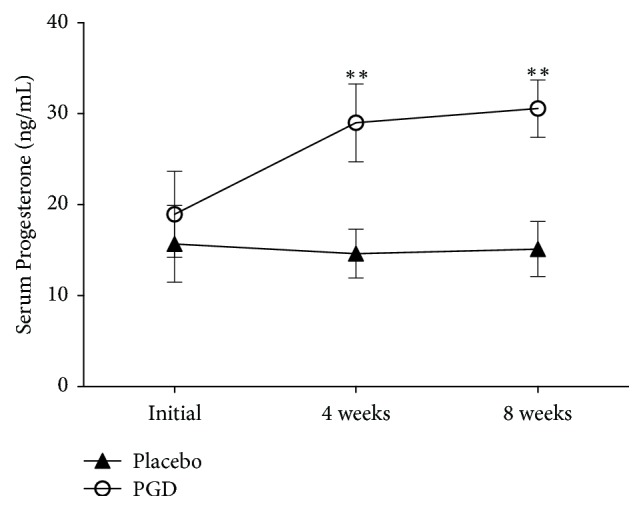
Mean levels of Progesterone in the study groups. Vertical bars represent standard deviations. Compared with placebo group, ^*∗∗*^*p* < 0.01.

**Figure 5:**
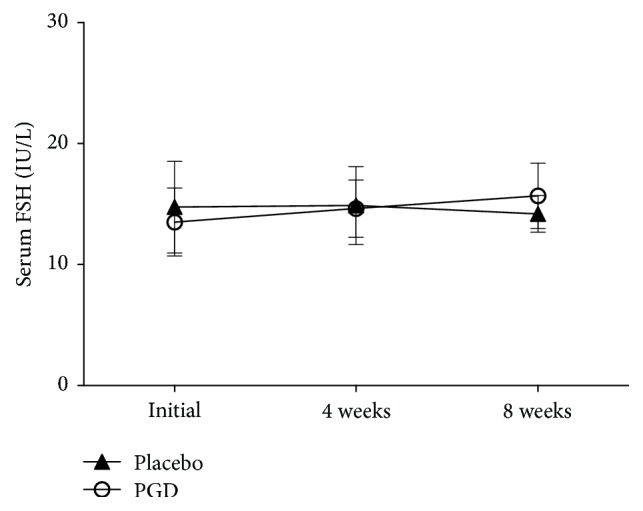
Mean levels of FSH in the study groups. Vertical bars represent standard deviations.

**Figure 6:**
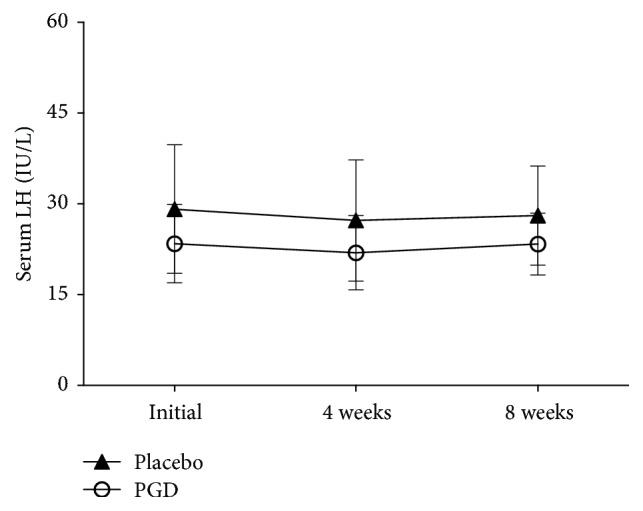
Mean levels of LH in the study groups. Vertical bars represent standard deviations.

Figures 2–6 represent levels of PRL, Estradiol, Progesterone, Follicle-stimulating hormone (FSH), and Luteinizing hormone (LH) at three different time points (initial, weeks 4 and 8). There were no differences in all hormones between these 2 groups at the beginning of the study. Levels of PRL have statistical significance at week 4 (placebo: 45.75 ± 5.57 ng/mL, PGD: 26.74 ± 4.03 ng/mL, *p* = 0.00) and week 8 (placebo: 45.27 ± 4.53 ng/mL, PGD: 25.00 ± 4.31 ng/mL, *p* = 0.00). Levels of Estradiol have no statistical significance at week 4 (placebo: 56.31 ± 25.95 pg/mL, PGD: 71.68 ± 20.61 pg/mL, *p* = 0.06) but have statistical significance at week 8 (placebo: 52.73 ± 24.65 pg/mL, PGD: 70.89 ± 17.53 pg/mL, *p* = 0.02). Levels of Progesterone have statistical significance at week 4 (placebo: 14.63 ± 2.68 ng/mL, PGD: 29.00 ± 4.29 ng/mL, *p* = 0.00) and week 8 (placebo: 15.13 ± 3.02 ng/mL, PGD: 30.58 ± 3.15 ng/mL, *p* = 0.00). Levels of FSH have no statistical significance at week 4 (placebo: 14.88 ± 3.22 IU/L, PGD: 14.63 ± 2.36 IU/L, *p* = 0.80) and week 8 (placebo: 14.20 ± 1.52 IU/L, PGD: 15.68 ± 2.71 IU/L, *p* = 0.07). Levels of LH have no statistical significance at week 4 (placebo: 27.25 ± 10.03 IU/L, PGD: 21.95 ± 6.13 IU/L, *p* = 0.08) and week 8 (placebo: 28.07 ± 8.19 IU/L, PGD: 23.37 ± 5.08 IU/L, *p* = 0.06).

### 3.3. Biochemical Indexes

The biochemical indexes of patients at week 8 in the study are shown in Table 2. There are no significant differences in hepatorenal function, blood glucose, blood lipid, and TSH between the two groups.

### 3.4. Treatment Emergent Symptom Scale (TESS)

After 8 weeks' treatment, the incidence of adverse reactions in TESS (NIMH, 1973) was 3/15 in the placebo group and 5/19 in the PGD group, respectively. The difference between these two groups has no statistical significance (*X*_2_ = 1.11, *p* > 0.05).

### 3.5. Positive and Negative Syndrome Scale (PANSS) Scores and Improvement of Hyperprolactinemia-Related Symptoms

**Figure 7:**
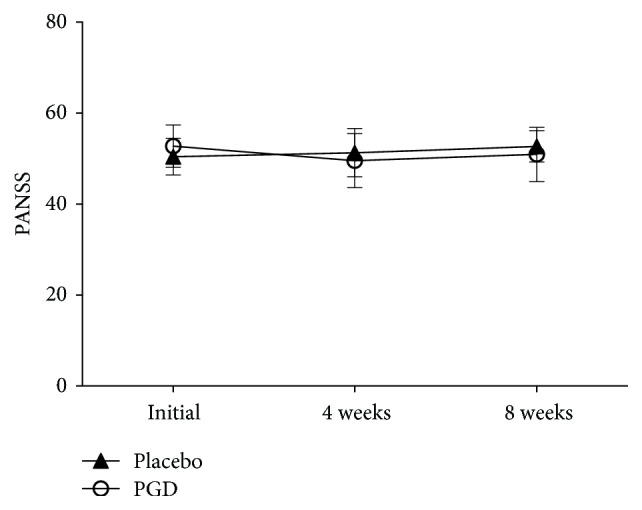
Mean PANSS in the study groups. Vertical bars represent standard deviations.

PANSS scores (Figure 7) have no statistical significance at the beginning of the study (placebo: 50.45 ± 4.05, PGD: 52.76 ± 4.65, *p* = 0.10), week 4 (placebo: 51.31 ± 5.29, PGD: 49.58 ± 5.93, *p* = 0.40), and week 8 (placebo: 52.73 ± 3.45, PGD: 50.95 ± 5.98, *p* = 0.85). At week 8, there was no improvement of hyperprolactinemia-related symptoms in the placebo group. On the contrary, 15 patients in the PGD group improved hyperprolactinemia-related symptoms in different aspects, among them, 2 cases in amenorrhea, 4 cases in abnormal menstruation, 3 cases in galactorrhea, 4 cases in reduced libido, and 2 cases in sexlessness.

## 4. Discussion

A great number of studies have shown that western medicine, such as Bromocriptine, Aripiprazole, and other drugs, will do antipsychotic-induced hyperprolactinemia more harm than good [23, 24]. Side effects of drugs are the most important factors leading to the withdrawal of medicine [25]. Therefore, the urgent priority is how to alleviate the side effects of antipsychotic drugs. Previous studies in cultured cells and animals suggested that PGD, a famous traditional Chinese decoction, would be effective in reducing the symptoms of antipsychotics-related hyperprolactinemia [16, 26, 27]. At present, clinical trials have successfully proved that the PGD can significantly reduce the symptoms of hyperprolactinemia caused by antipsychotic drugs [19]. However, the majority of the studies focused on the hyperprolactinemia induced by Risperidone, Sulpiride, and Olanzapine [28–30]; the data describing the Amisulpride-induced hyperprolactinemia were rare, which may be associated with its infrequent use in clinic. Meanwhile, the domestic investigation showed that Aripiprazole is effective in high PRL caused by Risperidone, but ineffective in that induced by Amisulpride [31, 32]. With the increase of schizophrenia patients treated with Amisulpride in our hospital in recent years, evidences have stated that Amisulpride-induced hyperprolactinemia has risen, especially in female patients. This status will result in many problems, such as reproductive development and bone density abnormality, which lead to the decline of compliance and disease recurrence. Hence, we selected some eligible patients to participate in this study.

The PGD contained the following: 60 g Paeoniae Radix Alba and 30 g Glycyrrhiza Radix Et Rhizoma. According to previous studies on the treatment of Qi-deficiency and blood-stasis syndrome, the ratio of Paeoniae Radix Alba to Glycyrrhiza Radix Et Rhizoma in PGD should be 1 : 1 [33]. However, in the treatment of hyperprolactinemia, a large number of studies have suggested that the ratio of 2 : 1 may be better [34]. Therefore, we choose this medicine compatibility in our experiment. As a result, we found that the levels of PRL and Progesterone were significantly improved with the treatment of PGD after 4 weeks, and the levels of PRL, Estradiol, and Progesterone were significantly improved after 8 weeks. Hyperprolactinemia-related symptoms of 15 patients in different aspects have been improved. It suggested that the level of PRL may be related to the regulation of hormones, and the PGD may inhibit the secretion of PRL by regulating the hormones level [26]. However, some scholars said that although the PGD can improve the symptoms of hyperprolactinemia caused by antipsychotics, the level of PRL did not change significantly [19]. The possible reason may be related to normalization of sex hormone dysfunction via dopaminergic action upon the hypothalamic-pituitary-gonadal axis [35, 36]. So, such a result we got may be associated with the medicine of Amisulpride we chose. Amisulpride has a strong affinity for D2 receptors, and their dissociation rate is much too slow [37, 38]. Amisulpride has a high selectivity to the central nervous system, affecting the mesolimbic system. While the rate of Amisulpride passing through the blood brain barrier is too low, the possessing rate to hypophyseal dopamine receptor is significantly higher than that to the middle part of the limbic system [39], which increases the impact to serum PRL in patients. This may be the difference between Amisulpride and other antipsychotic medications. Of course, our conclusion should be confirmed by further studies because we have not verified that PGD can change the level of PRL by regulating D2 receptor.

Considering that the high dose of PGD may increase the side effects to patients, our study detected the liver and kidney function, blood lipids, blood glucose, and thyroid hormone, combined with the TESS to assess the safety of the medication. Fortunately, the parameters above did not change too much after 8 weeks' treatment in PGD group, and the TESS did not show overt side effects. Although some extrapyramidal reactions appeared, they were controlled easily by using trihexyphenidyl hydrochloride. The improvement of corresponding symptoms by traditional Chinese medicine has given the patients confidence to finish this study, resulting in such low expulsion rate.

From all above, when western medicine cannot relieve Amisulpride-induced hyperprolactinemia, the PGD may work. This traditional Chinese decoction is simple and effective, with low side effect, which is worth being popularized in clinic. Further studies should be carried out to investigate the mechanism of how does it affect hyperprolactinemia.

## Figures and Tables

**Table 1:** Baseline characteristics of participant.

Parameter	Placebo (*n* = 20)	PGD (*n* = 21)	*p*
Age (years)	28.55 ± 5.24 (25.28–31.12)	28.42 ± 3.95 (26.95–29.36)	0.93
Duration of the illness (months)	16.75 ± 8.30 (11.72–20.41)	17.14 ± 7.15 (15.57–20.11)	0.87
Number of psychotic relapses	8 (40%)	9 (42.9%)	0.8
Amisulpride treatment duration (months)	6.6 ± 3.28 (4.50–6.96)	7.09 ± 2.36 (6.54–8.08)	0.58
Amisulpride dosage	0.88 ± 0.24 (0.78–1.04)	0.85 ± 0.26 (0.76–0.92)	0.78
Hyperprolactinemia-related symptoms			0.98
Amenorrhea	5 (25%)	5 (23.8%)	
Abnormal menstruation	5 (25%)	4 (19%)	
Galactorrhea	4 (20%)	5 (23.8%)	
Reduced libido	4 (20%)	4 (19%)	
Sexlessness	2 (10%)	3 (14.3%)	
PRL (ng/mL)	46.25 ± 5.43 (43.08–48.78)	47.62 ± 5.16 (45.63–48.90)	0.41
*Hormone assays*			
Estradiol (pg/mL)	54.25 ± 27.98 (37.53–73.14)	59.67 ± 26.21 (49.55–66.87)	0.53
Progesterone (ng/mL)	16.10 ± 4.01 (13.77–17.43)	16.57 ± 3.59 (15.41–17.54)	0.69
FSH (IU/L)	14.75 ± 3.80 (13.60–17.74)	13.52 ± 2.82 (12.53–14.41)	0.25
LH (IU/L)	27.15 ± 10.62 (22.45–28.03)	23.43 ± 6.48 (20.72–24.96)	0.19
Initial PANSS score	50.45 ± 4.04 (48.45–52.88)	52.76 ± 4.65 (51.12–54.24)	0.08
Positive subscale	19.85 ± 3.95 (18.22–21.38)	20.52 ± 3.31 (19.12–21.20)	0.60
Negative subscale	12.00 ± 3.34 (10.87–14.06)	13.71 ± 3.68 (12.55–15.03)	0.09
General subscale	18.60 ± 2.64 (16.95–19.84)	18.52 ± 4.48 (17.23–20.24)	0.95
*Biochemical indexes*			
ALT (U/L)	33.85 ± 12.93 (27.03–42.17)	36.33 ± 10.46 (32.06–38.99)	0.50
AST (U/L)	29.55 ± 10.67 (25.44–37.49)	33.52 ± 7.68 (30.39–34.87)	0.18
BUN (mmol/L)	5.47 ± 2.26 (4.61–7.15)	5.34 ± 1.51 (4.78–5.78)	0.83
CRE (*μ*mol/L)	69.24 ± 15.09 (60.98–79.78)	68.77 ± 10.66 (64.18–70.83)	0.91
TG (mmol/L)	2.92 ± 1.70 (2.04–3.96)	2.89 ± 1.51 (2.24–3.17)	0.95
GLU (mmol/L)	4.76 ± 0.76 (4.26–5.00)	4.98 ± 0.68 (4.74–5.19)	0.32
TSH (uIU/mL)	6.45 ± 2.36 (5.14–8.07)	7.73 ± 1.75 (7.08–8.27)	0.054

Data given as *n* (%) or mean ± standard deviation (95% CI (confidential intervals)); PRL = Prolactin; FSH = Follicle-stimulating hormone; LH = Luteinizing hormone; PANSS = Positive and Negative Syndrome Scale; ALT = alanine aminotransferase; AST = aspartate transaminase; BUN = blood urea nitrogen; CRE = creatinine; TG = triglyceride; GLU = blood glucose; TSH = thyroid stimulating hormone; NS = nonsignificant.

**Table 2:** Biochemical indexes after 8 weeks.

Parameter	Placebo (*n* = 15)	PGD (*n* = 19)	*p*
ALT (U/L)	38.00 ± 9.70 (32.63–43.37)	36.32 ± 10.04 (33.06–39.57)	0.62
AST (U/L)	32.27 ± 9.64 (26.93–37.61)	35.32 ± 7.97 (32.73–37.90)	0.32
BUN (mmol/L)	5.93 ± 2.13 (4.75–7.11)	5.59 ± 1.09 (5.24–5.94)	0.58
CRE (*μ*mol/L)	71.47 ± 14.91 (63.21–79.72)	65.91 ± 9.54 (62.82–69.00)	0.20
TG (mmol/L)	3.20 ± 1.46 (2.39–4.01)	2.85 ± 1.15 (2.48–3.23)	0.44
GLU (mmol/L)	4.72 ± 0.67 (4.35–5.10)	5.08 ± 0.58 (4.89–5.27)	0.32
TSH (uIU/mL)	6.41 ± 2.27 (5.15–7.66)	7.31 ± 1.81 (7.08–8.27)	0.21

Data given as *n* (%) or mean ± standard deviation (95% CI (confidential intervals)).

## Abbreviations

PGD: Peony-Glycyrrhiza Decoction
PRL: Prolactin
UPLC: Ultra Performance Liquid Chromatography
PANSS: Positive and Negative Syndrome Scale
CI: Confidential intervals
TESS: Treatment Emergent Symptom Scale.
